# An Experimental Study of the Frictional Properties of Steel Sheets Using the Drawbead Simulator Test

**DOI:** 10.3390/ma12244037

**Published:** 2019-12-04

**Authors:** Tomasz Trzepiecinski, Andrzej Kubit, Ján Slota, Romuald Fejkiel

**Affiliations:** 1 Department of Materials Forming and Processing, Rzeszow University of Technology, al. Powst. Warszawy 8, 35–959 Rzeszów, Poland; 2 Department of Manufacturing and Production Engineering, Rzeszow University of Technology, al. Powst. Warszawy 8, 35–959 Rzeszów, Poland; akubit@prz.edu.pl; 3 Institute of Technology and Material Engineering, Faculty of Mechanical Engineering, Technical University of Košice, Mäsiarska 74, 04001 Košice, Slovakia; jan.slota@tuke.sk; 4 Department of Mechanical Engineering, State School of Higher Vocational Education, Rynek 1, 38–400 Krosno, Poland; rfejkiel@wp.pl

**Keywords:** deep drawing, friction, mechanical engineering, steel sheet, sheet metal forming, tribology

## Abstract

This article presents the results of an experimental investigation of the frictional resistance arising in a drawbead during sheet metal forming. The frictional characterization of DC04 deep drawing quality steels commonly used in the automotive industry is carried out using a friction simulator. The effects of some parameters of the friction process on the value of the coefficient of friction have been considered in the experimental investigations. The friction tests have been conducted on different strip specimens, lubrication conditions, heights of drawbead and specimen orientations in relation to the sheet rolling direction. The results of drawbead simulator tests demonstrate the relationship that the value of the coefficient of friction of the test sheets without lubrication is higher than in the case of lubricated sheets. The lubricant reduces the coefficient of friction, but the effectiveness of its reduction depends on the drawbead height and lubrication conditions. Moreover, the effectiveness of the reduction of the coefficient of friction by the lubricant depends on the specimen orientation according to the sheet rolling direction. In the drawbead test, the specimens oriented along the rolling direction demonstrate a higher value of coefficient of friction when compared to the samples cut transverse to the rolling direction. The smaller the width of the specimen, the lower the coefficient of friction observed. The difference in the coefficient of friction for the extreme values of the widths of the specimens was about 0.03–0.05. The use of machine oil reduced the coefficient of friction by 0.02–0.03 over the whole range of drawbead heights. Heavy duty lubricant even reduced the frictional resistances by over 50% compared to dry friction conditions. The effectiveness of friction reduction by machine oil does not exceed 30%.

## 1. Introduction

Frictional resistances arising in sheet metal forming are a function of many metal forming conditions; i.e., sliding velocity, material properties of the workpiece and tools, temperature, lubrication conditions, contact pressures, real contact area, character of load and sheet topography [1,2,3]. Two main mechanisms that accompany the friction process in sheet metal forming are surface flattening and roughening of the workpiece asperities. Both of these mechanisms strictly influence the effectiveness of friction reduction by the lubricant [4,5]. Friction, wear and lubrication are discussed by Button [6] in general terms to describe friction models, wear mechanisms and lubrication regimes commonly found in metal forming. The review made by Seshachayulu et al. [7] summarizes the role of friction, and the applicability of friction laws and different tests in measuring the coefficient of friction in sheet metal forming (SMF). In sheet metal forming a workpiece of sheet material is plastically deformed and formed in a drawpiece [8]. The forming process is based on sheet deformation caused by the relative movement between the tools and sheet metal. Appropriate control of the contact conditions in this process allows one to reduce or eliminate the defects of drawpieces; i.e., crack formation, wrinkles and excessive springback [2,9].

Over recent decades, many friction tests have been developed to investigate friction on different areas of workpieces in SMF [10,11]. The effects of different test stands on the coefficient of friction have been studied by Recklin et al. [12]. The authors assumed that the dominant influence of contact area size on the friction coefficient was caused by different lubricant distribution over the contact area. The non-uniformity of the deformation of the drawpiece is mainly determined by the occurrence of friction forces at the interface of the deformed material and the tool. Former studies [13,14,15] showed that the surface properties of the tool have a significant influence on the friction and wear behaviour under lubricated conditions.

Friction is a crucial factor influencing the accuracy of the results of numerical simulations. Progress in simulation techniques and the advantages and disadvantages of numerical methods for simulating the sheet metal forming process are discussed by Ablat et al. [16]. The influence of surface roughness and strain rate on the results of simulations of sheet metal forming have been investigated by Sigvant et al. [17]. The authors concluded that non-uniform tool surface roughness resulting in a non-uniformity of friction might result in significant differences between the predicted behaviour compared to the experimental behaviour during tool try-out or part production.

Drawbeads are commonly used to control material flow in the specific regions of the drawpiece during the drawing operation in order to achieve the optimal forming of a part without cracks and wrinkles. The position and height of drawbeads assures the production of an optimal stamped part with a minimal usage of material. Several studies have been dedicated to the identification of the effect of the geometry of the drawbead on the value of the coefficient of friction. Trzepieciński and Lemu [2] conducted experimental and numerical investigations on the effect of sheet metal surface roughness, lubricant conditions and sample orientation on the value of the coefficient of friction in the drawbead region in sheet metal forming processes. The results have ascertained several relationships showing the effects of surface profile and lubrication on the value of the coefficient of friction. Ke et al. [18] experimentally investigated deep drawing quality steel sheets flowing through the drawbead. The investigations of pre-strained specimens showed that bending and unbending under tension had a significant effect; namely, increasing the percentage of material elongation. Schmid et al. [19] investigated the forming limit of DC04 sheet metal after passing a drawbead. They concluded that material formability is significantly reduced as an effect of the drawbead. Bassoli et al. [20] developed a drawbead simulator to measure the restraining force exerted on a sheet metal by the drawbead. The results show that some geometries of drawbead cause the restraining force to be almost insensitive to changes in friction conditions.

In recent decades, numerical methods have been used to analyze sheet metal deformation at the drawbead. Samuel [21] conducted finite element-based numerical simulations to determine the pull force, shear force and bending moment required to form sheet metal subject to plane strain at the drawbead. Billade and Dahake [22] analyzed the strain and thickness variations during the forming process by improving the drawbead design. Murali et al. [23] optimized the location of rectangular and circular drawbeads and analyzed the thickness variations during the cup drawing process. They concluded that rectangular drawbeads restrain the material more than circular drawbeads. Wang et al. [24] proposed a geometric model of a real drawbead and conducted a fully automated design process that was put into operation, including geometric modelling, finite element analysis, intelligent optimization of the drawbead geometry and die manufacturing. The proposed geometric drawbead improved by a priori heuristic parameter adjustment (PHPA) demonstrated a good consistency with the experimental results. Courvoisier et al. [25] proposed an analytical model of a drawbead whose results have been compared with finite element-based simulations and experimental results.

**Figure 1:**
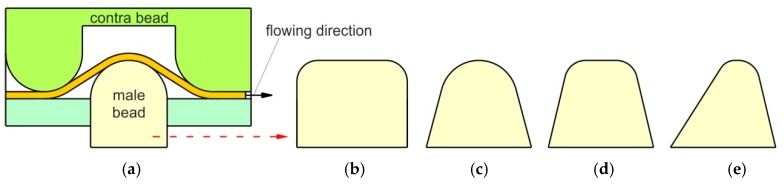
Geometries of drawbead cross-sections: (**a**) semi-circular, (**b**) rectangular, (**c**) triangular, (**d**) trapezoidal and (**e**) asymmetrical.

Some of the previous studies dealt with an equivalent drawbead model (EDM) [26]. Most EDMs developed represent the drawbead as an additional and constant drawbead restraining force. In real conditions, the sheet flow through the drawbead, and the plastic and frictional resistances depend on the stage of the process and strain hardening phenomenon of the sheet material. The material flow and restraining force can be controlled by an appropriate setting for both the geometry and position of the drawbead [27]. Drawbeads have the following geometrics: semicircular, square, triangular and trapezoid (Figure 1).

The basic method for analyzing sheet metal flow through the drawbead is using friction simulators which consist of three rollers representing the geometry of a real drawbead [2,18]. Passing the sheet metal through the drawbead simulator can be considered as the cycle of bending and unbending. So, the strain hardening phenomenon has a strong effect on the value of the restraining phenomenon. The width of the strip specimen affects the plastic and frictional resistance of the specimen flow through the drawbead simulator which, to the best of the authors’ knowledge, has not been indicated by other researchers. In the study presented herein, the frictional properties of deep drawing quality steel sheets were experimentally investigated using a drawbead simulator. The experimental system used represents a significant breakthrough in the analysis of frictional resistances that occur when pulling the sheet through a drawbead. The effects of some process parameters on the value of the coefficient of friction have been investigated. The friction tests have been conducted with different strip specimens, lubrication conditions, heights of drawbead, countersample roughnesses and specimen orientations related to the sheet rolling direction.

## 2. Materials and Methods

### 2.1. Material Characterisation

This article presents the results of an experimental investigation of frictional resistances using a drawbead simulator friction test. The research considered the frictional properties of 0.8 mm thick sheets of DC04 drawing quality steels that are commonly used in the automotive industry. The basic mechanical properties of the sheets tested were determined in the uniaxial tensile test according to the EN ISO 6892-1:2016-09, and the strain hardening parameters in the Hollomon equation *σ* = *K*·*ε^n^* have been listed in Table 1. The mechanical properties of the sheet metal were determined through tensile tests along three directions with respect to the rolling direction: 0°, 45° and 90°. Three specimens were tested for each cut direction.

**Figure 2:**
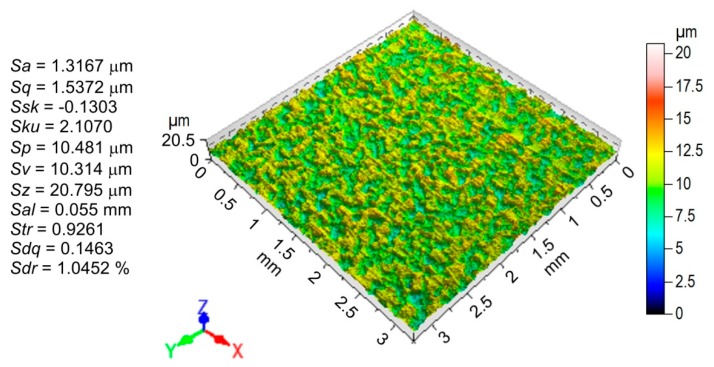
Basic roughness parameters and topography of the DC04 steel sheet tested.

The measurement of surface roughness parameters was carried out using the Talysurf CCI Lite 3D instrument. The main, standard 3D parameters (Figure 2) determined by this measurement according to the EN ISO 25178-6:2011 standard were the average roughness *Sa*, root mean square roughness parameter *Sq*, surface skewness *Ssk*, surface kurtosis *Sku*, highest peak of the surface *Sp*, maximum pit depth *Sv*, 10-point peak valley surface roughness *Sz*, auto-correlation length *Sal*, texture aspect ratio *Str*, root mean square gradient *Sdq* and developed interfacial area ratio *Sdr*. The morphology of the specimens after the friction tests was examined using a Hitachi S-3400N (Hitachi, Chiyoda, Japan) scanning electron microscope.

### 2.2. Experiment

**Figure 3:**
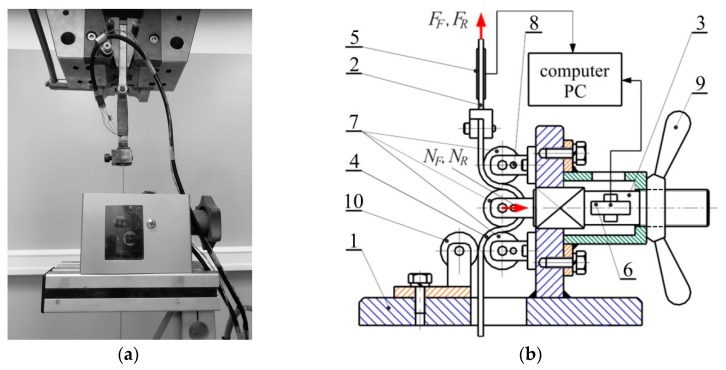
View (**a**) and model (**b**) of the drawbead simulator: 1—frame, 2—vertical tension cell, 3—horizontal load cell, 4—specimen, 5—vertical load cell, 6—horizontal load cell, 7—working rollers, 8—blocked pin, 9—nut and 10—supporting roller.

The curvature of the metallic sheet passing through the drawbead simulator (Figure 3a) was changed several times, the sheet was alternately bent and straightened. Two tests were required to determine value of the coefficient of friction. Firstly, the sheet strip was pulled by a system of fixed rollers. Next, the strip was pulled to flow over rotatable rollers. In the test, when pulling the strip, the values of the pulling forces *F_F_* and *F_R_*, and the clamping forces *N_F_* and *N_R_*, were measured (Figure 3b). The pulling force in this case may be connected with the deformation resistance of the sheet. The system of fixed rollers represents the total resistance of the sheet drawing through the drawbead. In the drawbead simulator, the sheet metal was pulled to flow between three cylindrical rollers of equal radii of 20 mm. The test material was cut along the rolling direction and transverse direction into 400 mm long.

The set of the friction test conditions were as follows:

- Surface roughness of rollers: *Ra* = 0.32, 0.63 and 1.25 mm;
- Friction conditions: dry friction, lubrication using machine oil LAN-46 (Orlen Oil) and lubrication using a chlorinated, honey-type, compound, heavy duty stamping oil—Heavy-Draw 1150 (Lamson Oil);
- Specimen orientations: 0° and 90° according to the sheet rolling direction;
- Strip widths *b*: 7, 14 and 20 mm;
- Drawbead heights *h*: 6, 12, 18 mm.

The physico-chemical properties of the oils used based on the manufacturer’s data sheets are as follows:

- LAN-46: density at 15 °C, *ρ* = 857 kg·m^−3^; kinematic viscosity at 40 °C, *η* = 43.9 mm^2^·s^−1^; viscosity index, *i_η_* = 94; flow temperature, −10 °C; flash point, *T_f_* = 232 °C;
- Heavy-Draw 1150 oil: density at 20 °C, *ρ* = 975 kg·m^−3^; viscosity at 40 °C, *η* = 1157 mm^2^·s^−1^; flash point *T_f_* = 277 °C.

**Figure 4:**
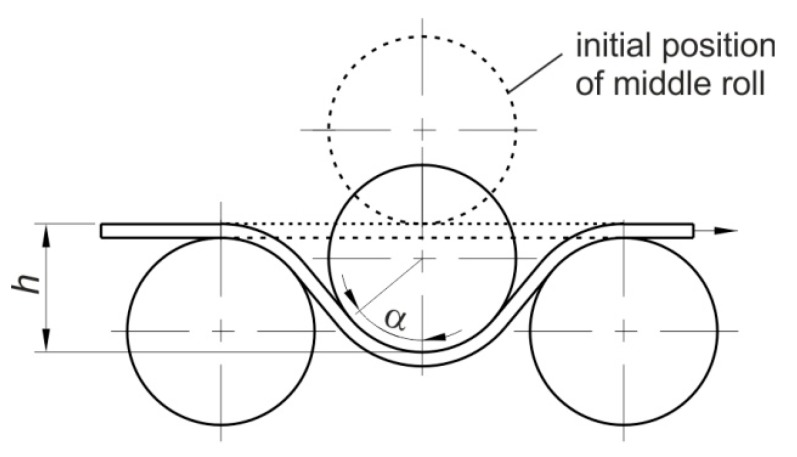
The geometrical parameters of the drawbead simulator.

When the wrap angle *α* is not equal to 90° (Figure 4) the value of the friction coefficient is determined using an Equation [28]:

(1) $$\mu =\frac{sin\alpha }{2\alpha }\;\times \;\frac{F_{F}-F_{R}}{N_{F}},$$

where *F_F_* is the pulling force obtained with the fixed rollers; *F_R_* is the pulling force obtained with the freely rotating rollers; *N_F_* is the normal force or clamping force obtained with the fixed beads; and *α* is the quarter contact angle of actual engagement of the strip over the middle roller.

**Figure 5:**
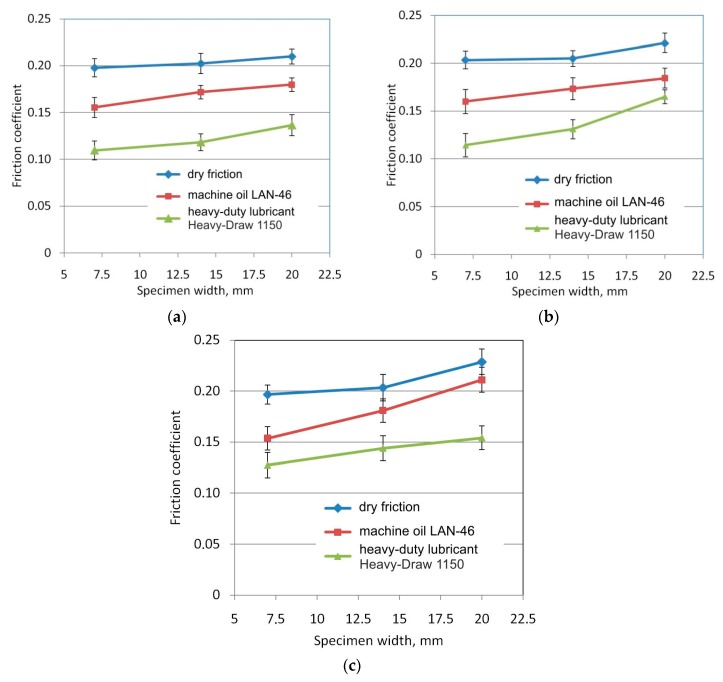
The effect of specimen width on the value of the coefficient of friction for the tests carried out at a drawbead height equal 12 mm and specimen orientation 0°, and for the following surface roughness values of rollers: 0.32 μm (**a**), 0.63 μm (**b**) and 1.25 μm (**c**).

The standard deviation (SD) was used to present the characteristics of sample data and to explain statistical analysis. The results presented in Figure 5 contains bars of ±SD.

## 3. Results and Discussion

### 3.1. The Effect of Specimen Width

**Figure 6:**
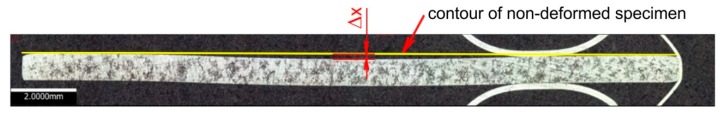
Deformation of the cross-section of a specimen with a width of 20 mm; friction conditions: *Ra* of rollers 1.25 μm; drawbead height 12 mm; dry friction.

The test specimens with the largest width of 20 mm showed the greatest frictional resistance (Figure 5). The smaller the width of the specimen, the lower the coefficient of friction. The difference in the coefficient of friction for the extreme values of the widths of the specimens was about 0.03–0.05. The influence of specimen width on the change of frictional resistance may be explained by the different method of deformation of the sheet metal strip, which was bent and straightened several times while passing through the drawbead. It has been observed that the sheet metal strips with a much larger width than the sheet thickness take a concave-convex shape (Figure 6), which can affect the real contact area and the difference in the mechanism by which roughness asperities are flattened along the specimen width. The tendency towards an increasing coefficient of friction was observed for all conditions of lubrication and orientation of the specimens. In the range of standard deviation of changes in the coefficient of friction, the influence of the surface roughness of the rollers on the value of this coefficient is negligible.

**Figure 7:**
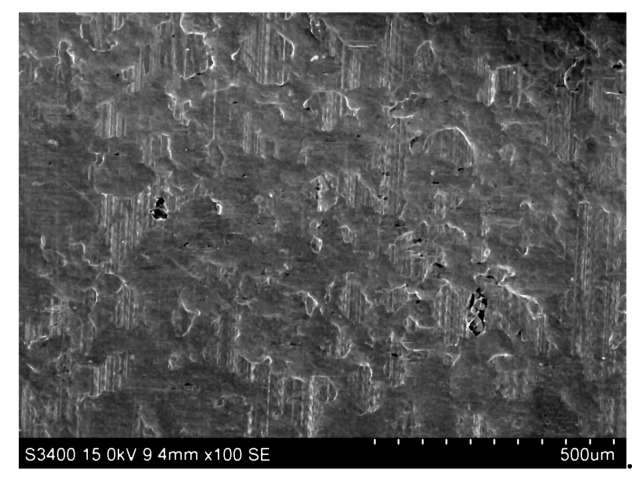
Scanning electron microscope micrograph of flattened surface asperities of a DC04 steel sheet tested with dry friction and a drawbead height 6 mm—middle part of the specimen width.

Drawbead height produces effects on the change in surface topography due to plastic strain, and consequently, on the two main friction mechanisms in dry friction conditions, flattening and adhesion. The strain hardening phenomenon of the sheet may lead to a different degree of plastic smoothing of asperities. Repeated plastic cyclic bending and straightening carried out on three cylindrical countersamples led to smoothening. This was produced by the flattening of the profile peaks and lifting of the valley grounds [18] and the phenomenon has been observed under both dry (Figure 7) and lubricated conditions. When a load was applied to a lubricated specimen surface, the asperities started to undergo plastic deformation. Therefore, the roughness valleys that entrap lubricant between the tool surface and the workpiece surface act as lubricant pockets [19]. In the case of the rollers with *Ra* = 1.25 μm and the highest drawbead height used *h* = 18 mm, a clear mechanism of ploughing appeared.

**Figure 8:**
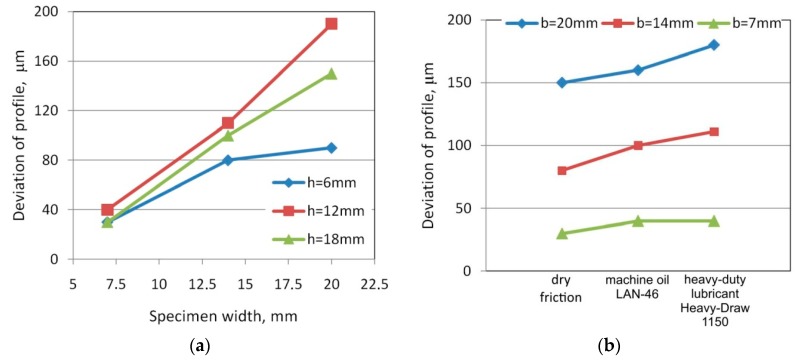
The effects of specimen width (**a**) and lubrication conditions (**b**) on the deviation of the cross-section of specimens; *Ra* of rollers 0.32 μm.

An increase in the sample width causes an increase in the deviation of the specimen profile Δx (Figure 6) from the original outline (Figure 8a). In the case of the drawbead height h = 12 mm, in the range of specimen widths used, a linear relationship was observed between specimen width and contour deviation (Figure 8a). The phenomenon of changing the profile is mainly the effect of repeated plastic cyclic bending and straightening around three cylindrical countersamples. The usage of lubricants increases the deformation of the cross-section of the specimens in the whole range of widths and drawbead heights. It was observed that the largest deviations occur during lubrication with Heavy Draw 1150 oil, and the smallest in dry friction conditions (Figure 8b). In contrast, the roughness of the rollers does not have a significant effect on the deformation of the cross-section of the samples. The deviation of the specimen profile for different roller roughnesses and under the same remaining friction conditions was not greater than 10 μm.

**Figure 9:**
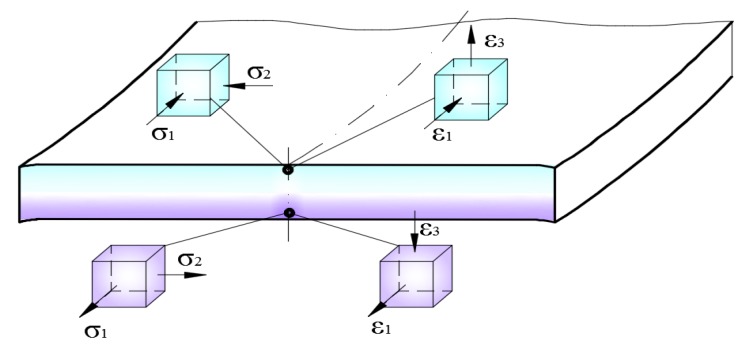
Stress and strain state during the bending of the sheet metal strip.

The sheet metal strip undergoes a change of curvature a few times as a result of cyclic bending and straightening when passing through the drawbead simulator. The specimen is subjected to dominant tensile stresses resulting from the interaction of the friction force on the surfaces of the rollers. It is known [9] that transverse components of stress arise when bending sheet metal strips with a ratio of width to thickness greater than about 4–5 (Figure 9), which distorts the process of deformation of the specimen along its width. Moreover, the cyclic bending and straightening of such a distorted specimen during the passing of the sheet metal through the subsequent rollers causes the sheet material to be subjected to the non-uniform strengthening process.

**Figure 10:**
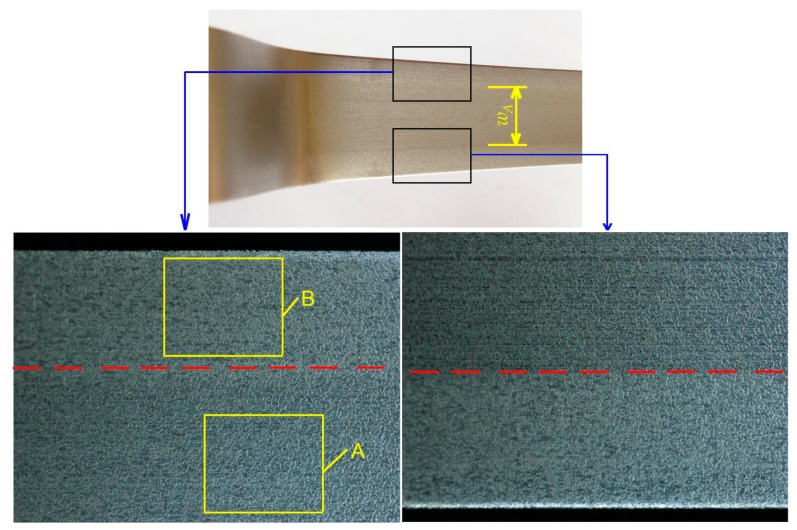
View of the specimen surface: specimen width 20 mm; drawbead height *h* = 18 mm; *Ra* of rollers 0.63 μm; machine oil lubrication.

An analysis of the surface of the samples after the friction process showed the presence of two clear zones in the specimens with widths 14 and 20 mm: a middle one with a width *w_A_* and lateral ones with different surface topographies (Figure 10). This fact can significantly affect the resistance of pulling the sheet through the drawbead, on the basis of which the coefficient of friction is determined.

**Figure 11:**
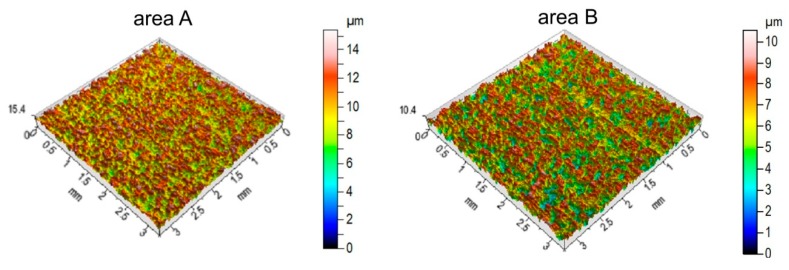
Comparison of isometric images of the sheet surface after the friction test for the areas A and B identified in Figure 10.

The measurements of roughness parameters carried out in two characteristic areas (A and B in Figure 10) show the different natures of surface topography changes in relation to the parameters measured on the surface before friction. A clearly different reduction in profile height was observed in the areas of the sample that were analyzed (Figure 11) compared to the initial surface (Figure 2). The asperities of surface topography were clearly flattened due to friction.

The values of selected amplitude, spatial, hybrid, volume and functional parameters measured after the friction test are presented in Table 2, Table 3, Table 4 and Table 5. The amplitude parameters, which considerably decreased their values as a result of the friction process, are *Sp* and *Sz* (Table 2). As a result of the friction process, the areal material ratio Smr increased significantly. For measurement in zone A, an increase of over 300 times—and for zone B, over 360 times—was noted (Table 3). The flattening of the surface asperities as a result of the interaction of the roller surface with the sheet metal caused the density of the peaks Spd to increase by factors of almost two (zone A) and almost four (zone B) compared to the original surface.

### 3.2. The Effect of Drawbead Height

**Figure 12:**
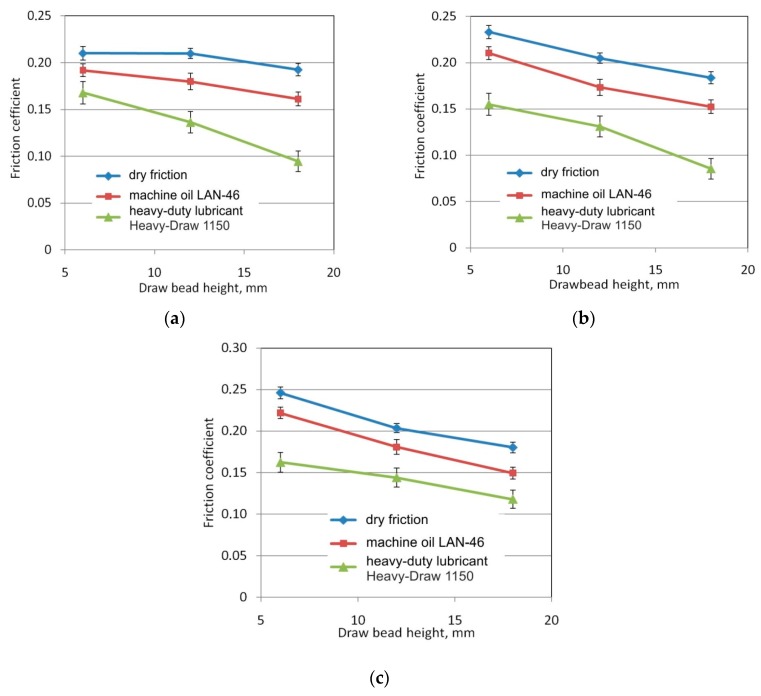
The effect of drawbead height on value of the coefficient of friction for the tests carried out on specimen width 20 mm, specimen orientation 0° and for the following surface roughnesses of rollers: (**a**) 0.32 μm, (**b**) 0.63 μm and (**c**) 1.25 μm.

An increase in the height of the drawbead resulted in an almost linear reduction in the coefficient of friction (Figure 12). With a low roughnesses of the rollers used, *Ra* = 0.32 μm (Figure 12a) and *Ra* = 0.63 μm (Figure 12b), the heavy duty stamping lubricant reduced the frictional resistance to the greatest extent. The use of machine oil reduced the coefficient of friction by 0.02–0.03 in the whole range of drawbead heights. The results show that the frictional resistance decreases proportionately with the height of the drawbead. An increase in the drawbead height increases the overall wrap angle of all rollers. This resulted in a significant increase in the resistance of sheet metal flow through the drawbead both in the case of rotating rollers and in the case of fixed rollers. The pulling resistance of the sheet metal strip through the system of fixed rollers in relation to flowing the strip through the rotatable rollers decreases so the coefficient of friction determined from Equation (1) decreases (Figure 12). Similar conclusions can be drawn for the widths of specimens of 7 and 14 mm and specimen orientation 90°.

### 3.3. The Effect of the Surface Roughness of a Roller

**Figure 13:**
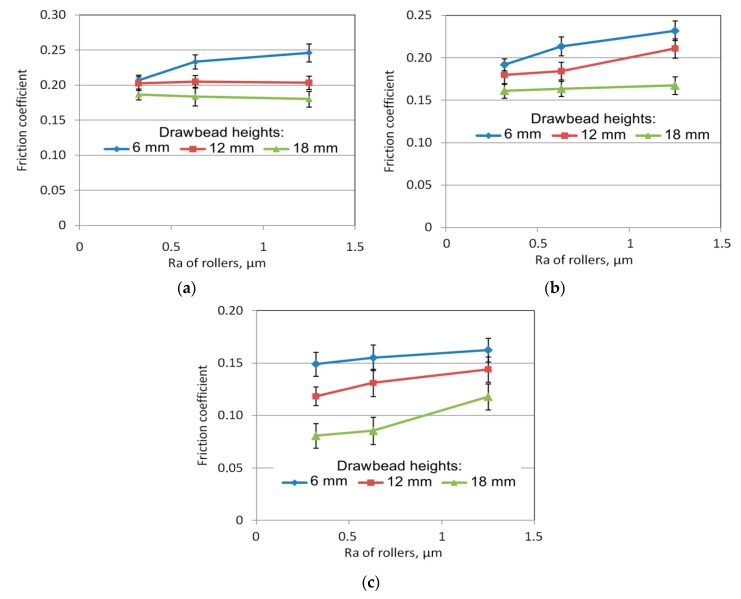
The effect of *Ra*s of rollers on the value of the coefficient of fiction for the tests carried out on specimen width 14 mm, specimen orientation 0° and for the following friction conditions: dry friction (**a**), machine oil (**b**) and heavy duty stamping lubricant (**c**).

The influence of roller roughness on the value of the frictional resistance is not unambiguous. In the case of dry friction conditions (Figure 13a), an increase of coefficient of friction with roughness of the rollers was only observed for the drawbead height of 6 mm. In machine oil lubrication conditions, this increase can also be detected with a drawbead height of 12 mm (Figure 13b). In other cases there is no significant effect of roller roughness on the value of the coefficient of friction, the value of which changes slightly. A different situation occurs with lubrication using heavy duty stamping lubricant (Figure 13c): for all drawbead heights an increase of the roughness of the rollers resulted in an increase in the coefficient of friction. A similar change in the frictional resistance was demonstrated in samples cut transversely to the sheet rolling direction.

### 3.4. The Effect of Specimen Orientation

**Figure 14:**
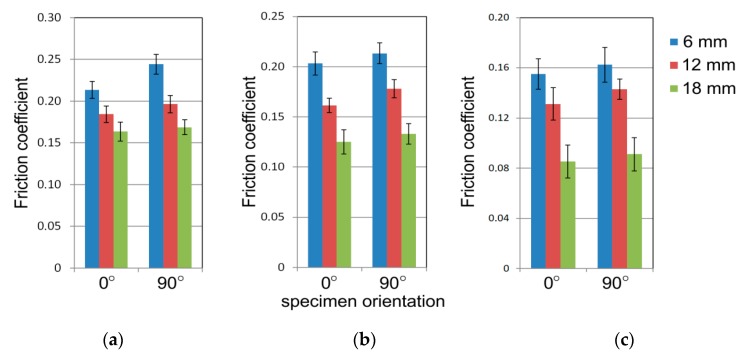
The effect of specimen orientation on the value of the coefficient of friction for the tests carried with an *Ra* of rollers of 0.63 μm and the following friction conditions: (**a**) 20 mm, (**b**) 7 mm and (**c**) 14 mm.

In general, the specimens cut transverse to the rolling direction exhibited a greater coefficient of friction than the specimens cut along the sheet rolling direction (Figure 14). This observation can be assigned to all the drawbead heights analysed. In the case of dry friction conditions, the difference between the coefficients of friction determined for both orientations was the most evident. In lubricating conditions, the differences were statistically insignificant.

### 3.5. The Effectiveness of Lubrication

To analyze the effect of lubricant type on the reduction of the value of the coefficient friction, the coefficient of effectiveness of lubrication was introduced:

(2) $$E_{c}=\frac{\mu_{d}-\mu_{l}}{\mu_{d}}\times 100\%,$$

where *μ_d_* and *μ_l_* are friction coefficients determined in dry friction and lubricated conditions. respectively.

**Figure 15:**
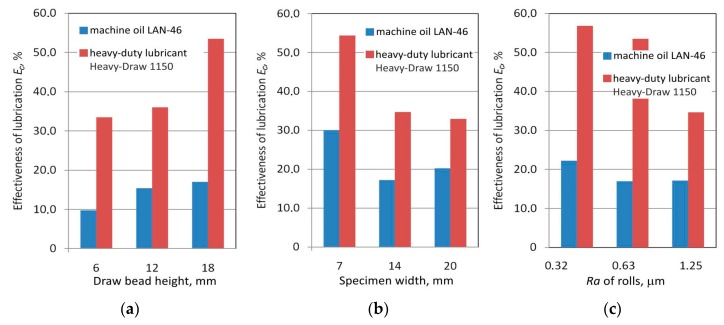
The effects of select parameters on the value of the coefficient of friction for the tests carried for specimen orientation 0° and the following friction conditions: specimen width 14 mm and *Ra* of rollers 0.63 μm; (**a**) drawbead height 18 mm and *Ra* of rollers 1.25 μm; and (**b**) drawbead height 18 mm and specimen width 14 mm (**c**).

Heavy duty lubricant even reduced the frictional resistances by over 50% compared to dry friction conditions (Figure 15). The effectiveness of friction reduction by machine oil did not exceed 30% (Figure 15b). For both lubrication conditions, an increase in the drawbead height caused a greater reduction of the coefficient of friction by the lubricant (Figure 15a). By contrast, an increase in the surface roughness of the rollers caused a decrease in lubrication effectiveness (Figure 15c). Two main mechanisms coexisted with lubrication in the friction process. The first mechanism is connected with the flattening of surface asperities caused by mechanical interaction of the tool-sheet interface. The second one supplied lubricant to the asperities contact zone by the oil pockets located in the surface roughness valleys. In the case of high surface roughness of the rollers, the first mechanism was dominant, so the effectiveness of lubrication was reduced (Figure 15c).

## 4. Conclusions

The results of friction tests ascertained several relationships showing the effect of friction process parameters on the value of the coefficient of friction during the flow of sheet metal through a drawbead during sheet metal forming. The following conclusions were drawn from the experimental research:

- Drawbead height and the lubrication conditions are significant factors that influence the coefficient of friction;
- The value of the coefficient of friction of the sheets tested without lubrication was higher than in the case of the lubricated sheets; this relationship was expected; the use of machine oil reduced the coefficient of friction by 0.02–0.03 across the whole range of drawbead heights;
- An increase in specimen width causes an increase in the value of the coefficient of friction; the difference in the coefficient of friction for the extreme values of the widths of the specimens was about 0.03–0.05;
- Lubricants reduce the coefficient of friction but the intensity of their action depends on the drawbead height and lubrication conditions; heavy duty stamping lubricant reduces the coefficient of friction much more than machine oil; heavy duty stamping lubricant reduces the frictional resistance to even less than half the figure observed in dry friction conditions;
- Heavy duty lubricant reduced the frictional resistances by over 50% compared to dry friction conditions; the effectiveness of friction reduction by machine oil does not exceed 30%;
- The flattening of the surface asperities as a result of the interaction of the roller surface with the sheet metal caused a significant increase in the areal material ratio; an increase of over 300 times was noted;
- The effect of the *Ra* of a roller on the degree of frictional resistance is not clear and depends on the drawbead height used and lubrication conditions.

In general, it was found that the effect of specimen orientation in relation to sheet rolling direction on the value of the coefficient of friction was not significant. There are two main properties of metallic sheets that influence the character of the specimen flowing through drawbead: plastic properties of material due to the crystallographic texture and directional texture of the sheet metal surface [29,30]. Further investigations are needed to clarify the relationship between these phenomena and their synergic action on the change in the frictional resistance.

## Figures and Tables

**Table 1:** Selected mechanical properties of the DC04 steel sheet (±standard deviation).

Specimen Orientation	Yield Stress *R_p0.2_* (MPa)	Ultimate Tensile Stress *R_m_* (MPa)	Elongation *A_50_* (%)	Strengthening Coefficient *K* (MPa)	Strain Hardening Exponent *n*
0°	184 ± 3.0	303 ± 6.2	23 ± 0.6	490 ± 5.84	0.20 ± 0.003
45°	193 ± 0.5	314 ± 0.4	22 ± 0.2	489 ± 4.63	0.16 ± 0.002
90°	176 ± 0.5	296 ± 0.7	22 ± 0.3	465 ± 3.97	0.16 ± 0.002

**Table 2:** Selected amplitude parameters of surface roughness of the specimen: width 20 mm; drawbead height *h* = 18 mm; *Ra* of rollers 0.63 μm; machine oil lubrication.

Source of Measurement	*Sa* (μm)	*Sq* (μm)	*Ssk*	*Sku*	*Sp* (μm)	*Sv* (μm)	*Sz* (μm)
original surface	1.32	1.54	−0.13	2.11	10.5	10.3	20.80
A	1.38	1.65	0.20	2.32	5.25	10.2	15.41
B	1.33	1.55	−0.07	2.03	4.35	6.2	10.55

**Table 3:** Selected functional, spatial and hybrid parameters of surface roughness of the specimen: width 20 mm; drawbead height *h* = 18 mm; *Ra* of rollers 0.63 μm; machine oil lubrication.

Source of Measurement	*Smr* (%)	*Smc* (μm)	*Sal* (mm)	*Str*	*Std* (°)	*Sdq*	*Sdr* (%)
original surface	0.00029	2.00	0.055	0.93	178.5	0.15	1.05
A	0.088	2.30	0.056	0.86	178.5	0.15	1.14
B	0.104	2.08	0.055	0.89	1.5	0.14	1.03

**Table 4:** Selected functional and spatial parameters of surface roughness of the specimen: width 20 mm; drawbead height *h* = 18 mm; *Ra* of rollers 0.63 μm; machine oil lubrication.

Source of Measurement	*Vm* (μm^3^/μm^2^)	*Vv* (μm^3^/μm^2^)	*Vmp (*μm^3^/μm^2^)	*Vmc (*μm^3^/μm^2^)	*Vvc (*μm^3^/μm^2^)	*Vvv (*μm^3^/μm^2^)
original surface	0.036	2.03	0.036	1.62	1.90	0.13
A	0.064	2.36	0.064	1.64	2.24	0.12
B	0.036	2.11	0.036	1.62	1.98	0.13

**Table 5:** Selected feature parameters of surface roughness of the specimen: width 20 mm; drawbead height *h* = 18 mm; *Ra* of rollers 0.63 μm; machine oil lubrication.

Source of Measurement	*Spd* (1/mm^2^)	*Spc* (1/mm)	*Sda* (mm^2^)	*Sha* (mm^2^)	*Sdv* (μm^3^)	*Shv* (μm^3^)
original surface	54.33	77.3	0.019	0.019	4795	3790
A	106.4	78.4	0.010	0.009	1923	1389
B	207.6	91.5	0.005	0.005	440	339
